# Optic Neuritis in a 15-Year-Old Female Patient: A Case Report and Literature Review

**DOI:** 10.7759/cureus.53222

**Published:** 2024-01-30

**Authors:** Leah Chan, Nicholas Pereira

**Affiliations:** 1 School of Medicine, Saint James School of Medicine, Anguilla, AIA; 2 Pediatrics, South Texas Health System Children’s, Edinburg, USA

**Keywords:** neuro-ophthalmology, pediatric, dyschromatopsia, acute vision loss, painful vision loss, optic nerve enhancement, multiple sclerosis and other demyelinating disorders, pediatric optic neuritis

## Abstract

Optic neuritis represents a rare clinical entity leading to vision loss within the pediatric population. While not an urgent or life-threatening condition, optic neuritis may serve as a manifestation of various systemic diseases. When pediatric patients present with complaints of vision loss, it is imperative to consider optic neuritis as a potential differential diagnosis. This prompts further investigation to exclude systemic diseases capable of causing substantial morbidity. This article details a case report involving optic neuritis in a 15-year-old Hispanic female, outlining the investigation and management approach. Additionally, a review of the literature identifies six recent case reports documenting pediatric optic neuritis within the last year.

## Introduction

Optic neuritis, characterized by inflammation of the optic nerve, typically manifests as acute unilateral vision loss, eye pain, and dyschromatopsia. It can occur as an isolated event or as a manifestation of systemic conditions, including infection, autoimmunity, paraneoplastic disorders, granulomatous disease, or demyelination disorders [1]. This condition most commonly affects young, healthy Caucasian females below the age of 50, often being associated with multiple sclerosis (MS). Notably, optic neuritis serves as the initial symptom of MS in approximately 20% of cases [1,2]. While the estimated annual incidence of optic neuritis is five per 100,000, its occurrence in the pediatric population is significantly lower, ranging between 0.15 and 0.57 per 100,000 [3].

Pediatric optic neuritis, particularly in prepubertal children, is more likely to present bilaterally with more severe vision loss and optic nerve swelling compared to its adult counterpart [4,5]. Similar to optic neuritis in adults, pediatric cases exhibit a higher prevalence in females, except in prepubertal children where the incidence is equal between both sexes. Optic neuritis in children can be associated with systemic or demyelinating disorders such as systemic lupus erythematosus (SLE), granulomatous disease, MS, neuromyelitis optica (NMO), or myelin oligodendrocyte glycoprotein (MOG) antibody-associated disease [1,2]. However, in children, it is most frequently correlated to parainfectious or postvaccination processes [3].

Diagnosis of optic neuritis involves comprehensive ophthalmic, neurologic, and systemic examinations, coupled with MRI. Brain and orbital MRI with gadolinium are invaluable in investigating atypical presentations, as seen in pediatric optic neuritis [1,4,6]. Treatment approaches for pediatric optic neuritis parallel those for adults, involving close observation or a three to five-day course of intravenous steroids based on case severity [1,3,4]. Refractory cases may necessitate intravenous immunoglobulin G (IgG) or plasma exchange [6]. Generally, outcomes are optimistic, with good recovery of visual acuity, although occasional irreversible injury, functional decline, and lingering deficits may impact vision-related quality of life [1,2,5].

## Case presentation

A previously healthy 15-year-old Hispanic female was admitted to the pediatric unit by her primary care physician due to optic neuritis requiring treatment, pain management, and further investigation. The patient’s chief complaint was worsening pain and decreased vision in the right eye over eight days. Four days after the onset of symptoms, the pain began radiating from the right eyeball to the right eyebrow. The patient reported that the pain was most intense during superior and lateral eye movements but was alleviated in a dark room. Upon examination, the patient described an inferior altitudinal visual defect, characterizing the inferior half of her vision in the right eye as moderately blurry and the superior half as mildly blurry. Within the hospital setting, the absence of an ophthalmic perimeter posed a constraint, and a formal visual field plot could not be acquired. She also noted difficulty perceiving colors and details but denied any changes in visual acuity in the left eye. The primary care physician promptly referred her to an ophthalmologist who diagnosed optic neuritis in the right eye, recommending immediate inpatient treatment and further investigation to rule out MS specifically.

The patient and her family had no known family history of optic neuritis or related conditions and denied recent trauma to the head or neck, illness, or vaccination. Additionally, the patient denied other associated neurological symptoms such as drowsiness, weakness, or alterations in the sensorium.

During the physical examination, all vital signs were within normal ranges. The patient remained conscious, cooperative, and well-oriented to time, person, and place. Her body temperature was 98.6°F, heart rate was 75 per minute, respiratory rate was 18 cycles per minute, and blood pressure was 99/62 mmHg. No signs of pallor, icterus, clubbing, cyanosis, edema, or lymphadenopathy were observed.

Examination of the right eye revealed a decreased pupillary light reaction and a relative afferent pupillary defect. Fundoscopic examination indicated the presence of papillitis and disc pallor in the right eye. No other neurological deficits were noted, and findings from the systemic examination, including the left eye, were normal. Laboratory and toxicology reports were unremarkable, except for a mildly elevated C-reactive protein level of 0.43 mg/dL (Table 1).

**Table 1:** Laboratory investigations at the time of presentation. All indices are within the normal limits except for CRP, which is mildly elevated at 0.43 mg/dL.

Test report	Result	Normal value	Unit
General chemistry			
Glucose	129 (high)	60–100	mg/dL
Sodium	138	130–147	mmol/L
Potassium	3.6	3.5–5.1	mmol/L
Chloride	109 (high)	95–108	mmol/L
Carbon dioxide	26	20–30	mmol/L
Blood urea nitrogen	12	2–20	mg/dL
Creatine	0.9	0.5–1	mg/dL
Calcium	8.9	8.4–10.2	mg/dL
Albumin	3.5	3.5–5.6	g/dL
Total protein	7.4	6–8	g/dL
Total bilirubin	0.20	<1.5	mg/dL
Alkaline phosphatase	92	70–320	IU/L
Aspartate aminotransferase	<3 (low)	5–30	IU/L
Alanine aminotransferase	19	5–55	IU/L
Estimated glomerular filtration rate	72.89	90–140	mL/minute/1.73m^2^
C-reactive protein	0.43 (high)	<0.3	mg/dL
General hematology			
White blood cells	9	4–13.5	×10^3^ cells/mm^3^
Red blood cells	3.85	4–5.5	×10^6^ cells/mm^3^
Hemoglobin	11.7 (low)	12–15	g/dL
Hematocrit	34.8 (low)	35–45	%
Mean corpuscular volume	90.5	78–95	fL
Mean corpuscular hemoglobin	30.4	26–32	pg
Mean corpuscular hemoglobin concentration	33.6	31–37	g/dL
Red cell distribution width - coefficient of variation	13.9	<14.5	%
Platelets	192	150–450	×10^3^ cells/µL
Mean platelet volume	10.3	9.4–12.4	fL
Neutrophil	69.4	35–80	%
Lymphocyte	24 (low)	25–45	%
Monocyte	5.7	3–6	%
Eosinophil	0.5	0–3	%
Basophil	0.4	0–1	%
Neutrophil	6.2	1.8–8	×10^3^ cells/µL
Lymphocyte	2.2	1.5–6.5	×10^3^ cells/µL
Monocyte	0.5	0.4–2	×10^3^ cells/µL
Eosinophil	0	0–0.8	×10^3^ cells/µL
Basophil	0	0–0.1	×10^3^ cells/µL
Sedimentation rate	19	<20	mm/hour
Pregnancy test	Negative		
Toxicology			
Urine amphetamine	Negative		
Urine barbiturates	Negative		
Urine benzodiazepine	Negative		
Urine cannabinoids	Negative		
Urine cocaine	Negative		
Urine opiates	Negative		
Urine phencyclidine	Negative		
Urine tricyclic antidepressants	Negative		

An infectious profile was conducted as part of the diagnostic process to exclude potential infectious causes of optic neuritis. The results from these labs were also within normal ranges (Table 2).

**Table 2:** Infectious disease immunology and serology laboratory results. No infection was detected in the serum.

Infectious disease immunology/Serology	Result
Herpes simplex virus 1	Not detected
Herpes simplex virus 2	Not detected
Herpes simplex virus 3	Not detected
*Escherichia coli* K1	Not detected
Cytomegalovirus	Not detected
Enterovirus	Not detected
Human parechovirus	Not detected
Varicella-zoster virus	Not detected
Listeria monocytogenes	Not detected
Streptococcus agalactiae	Not detected
Streptococcus pneumoniae	Not detected
Neisseria meningitidis	Not detected
Cryptococcus neoformans/gattii	Not detected
Haemophilus influenzae	Not detected

**Figure 1:**
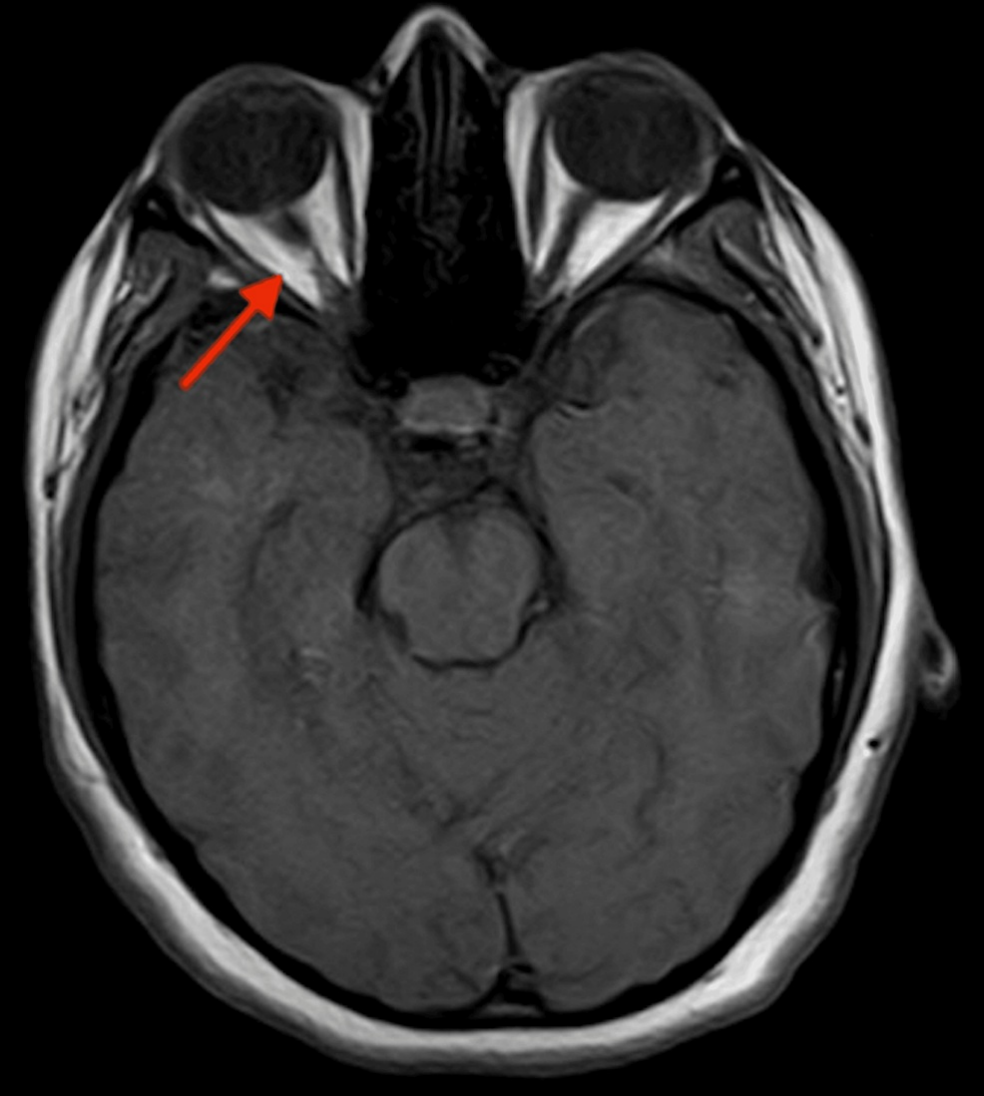
T1-weighted axial MRI with contrast of the skull and orbits showing enhancement of the right optic nerve. The image was negative for any other intracranial pathologies including white matter hyperintensities.

The decision was taken to involve neurology for a more in-depth investigation to rule out MS and other demyelination disorders. Subsequent imaging, including an MRI of the brain and orbits, revealed no intracranial pathology or white matter hyperintensity. However, the imaging demonstrated increased T1 signal intensity and enhancement throughout the right optic nerve on post-contrast images. Disconjugate gaze was also observed, confirming the diagnosis of optic neuritis (Figure 1).

A lumbar puncture was performed to assess for IgG oligoclonal bands, anti-aquaporin 4 antibodies, and anti-MOG antibodies. The results from this procedure also yielded normal results, assisting in the elimination of MS, NMO, or MOG antibody-associated disease as possible causes (Table 3).

**Table 3:** Cerebrospinal fluid lab results. All indices are normal except for minimal red blood cells due to traumatic tap during sample collection and no evidence of IgG oligoclonal bands, anti-aquaporin 4 antibodies, or anti-myelin oligodendrocyte glycoprotein antibodies were found. CSF: cerebrospinal fluid; IgG: immunoglobin G

Test report	Result	Normal value	Unit
CSF cell count			
CSF appearance	Clear	Clear	
CSF color	Colorless	Colorless	
CSF red blood cells	2 (high)	Nil	Cells/mm^3^
CSF chemistry			
CSF glucose	67	50–80	mg/dL
CSF total protein	21.05	15–40	mg/dL
IgG oligoclonal bands	Negative	Negative	
Anti-aquaporin 4 antibodies	Negative	Negative	
Anti-myelin oligodendrocyte glycoprotein antibodies	Negative	Negative	

The patient was treated with intravenous methylprednisone (1 g/day for five days) in the hospital. She reported daily improvement in both visual acuity and pain. The patient’s vision was clear on day five of treatment and was discharged in stable condition with oral prednisone to taper the dose of steroids over 15 days (50 mg once per day for three days, followed by 25 mg once per day for three days, 12.5 mg once per day for three days, and 12.5 mg once every other day for three days). The patient was instructed to follow up with her ophthalmologist within the next two days, and her primary care physician within three days, and to return to the hospital should her symptoms return or worsen.

## Discussion

This case presents a rare instance of optic neuritis in a pediatric patient. The typical presentation of pediatric optic neuritis mirrors that of adults, characterized by symptoms such as eye pain, dyschromatopsia, and significant visual acuity deficits. Common physical examination findings include a diminished pupillary light reflex and a relative afferent pupillary defect in the affected eye [7].

The diagnosis of optic neuritis involves a combination of physical examination, imaging, and laboratory investigations. In this case, the patient exhibited overall normal laboratory results, distinctive radiologic features of optic neuritis on MRI, and physical examination findings consistent with the condition.

Of note, the identification of papillitis on the fundoscopic examination and the patient’s description of an inferior altitudinal visual field defect is considered rare within the context of optic neuritis. Specifically, fewer than two-thirds of optic neuritis cases manifest with papillitis [1], and the occurrence of altitudinal visual field abnormalities in optic neuritis is even more infrequent. While they have been associated with optic neuritis in the past, altitudinal visual field defects are predominantly characteristic of ischemic optic neuropathy. Within the Optic Neuritis Treatment Trial, only 8% of the affected eyes exhibited altitudinal visual field abnormalities [8].

During the hospitalization period, a more extensive workup could have included laboratory vasculitis profiles to explore the presence of anti-neutrophil cytoplasmic antibodies, anti-nuclear antibodies, angiotensin-converting enzyme, and imaging of the chest to help rule out systemic conditions such as SLE, sarcoidosis, or other granulomatous diseases. In this instance, the etiology of the patient’s condition remained undetermined.

Accurate diagnosis and further investigation are crucial as several incidences of optic neuritis are associated with demyelinating diseases or systemic conditions. Optic neuritis in the pediatric population is more likely to be an initial manifestation of acute disseminated encephalomyelitis [6]. More studies are needed on the etiology and pathophysiology of optic neuritis in pediatric patients that can result in more effective prevention, diagnosis, and management for better patient outcomes.

In November 2023, a literature review was conducted using resources such as PubMed and EBSCO electronic databases. The keywords “pediatric,” “optic neuritis,” “child,” and “children” with the use of the Boolean operators “AND,” “OR,” and “NOT” were used to identify relevant studies discussing optic neuritis in children. The following inclusion criteria were applied: (1) a scholarly reviewed journal/source such as PubMed, (2) recent case studies published within the last year at the beginning of the date of research, (3) articles published in the English language, and (4) articles that included human participants only. The following exclusion criteria were applied: (1) participants over the age of 17, and (2) any study other than case reports. In total, only six case reports of optic neuritis in a pediatric patient have been published in the last year (Table 4).

**Table 4:** Demographics of case reports of imaging-confirmed pediatric optic neuritis in the last year.

Patient	Author	Age	Gender	Associated systemic disease/ Etiology
1	Chaudhary et al. 2023 [9]	6	M	Post-typhoid fever
2	Alassaf et al. 2023 [10]	15	F	Type 1 diabetes mellitus
3	Kadam et al. 2023 [11]	8	M	Myelin oligodendrocyte glycoprotein antibody disease
4	Diane et al. 2023 [12]	8	F	Post-upper respiratory infection
5	Rana et al. 2023 [13]	16	M	Demyelinating disease
6	Maran et al. 2023 [14]	13	M	Myelin oligodendrocyte glycoprotein antibody disease

In contrast to the average, there was a male predominance (67%) of reported cases of pediatric optic neuritis. Table 4 documents the demographics and reported cause of each case of optic neuritis within the last year. Of note, most cases were post-infectious [9,12] or related to MOG antibody disease [11,14]. All patients presented with decreased visual acuity [9-14]; however, not all patients presented with optic pain [9,10,14] and dyschromatopsia. In all six cases, each patient was treated with intravenous methylprednisone with a resolution of symptoms. In cases where the patient was followed up several months later, recurrence of disease [11] or long-term functional deficits were found upon examination [14]. Interestingly, both of the mentioned cases of optic neuritis were found to be caused by MOG antibody disease. However, other case studies did not report a follow-up examination; hence, relapse and long-term functional deficits could not be evaluated with other etiologies.

Future studies focusing on the long-term effects of optic neuritis in the pediatric population would be valuable. These studies can contribute significantly by reporting follow-up conditions of patients, specifically investigating whether any long-term visual deficits persist. Understanding the lasting impact of pediatric optic neuritis on visual function and quality of life is essential for informing comprehensive care strategies and improving outcomes.

## Conclusions

Optic neuritis, marked by eye pain, decreased visual acuity, and dyschromatopsia, is notably less common in the pediatric population compared to adults. While pediatric and adult optic neuritis share similarities in terms of diagnosis, treatments, and outcomes, there are notable differences in the primary causes. In the pediatric demographic, optic neuritis often develops following recent infections or vaccinations. A comprehensive investigation is crucial to rule out other potential causes, with a particular emphasis on excluding demyelinating and systemic diseases. In the presented case, despite examination and investigation, the etiology of the patient’s optic neuritis remained unidentified. However, prompt treatment led to a satisfactory resolution of symptoms and the restoration of visual acuity.
